# PPARγ Activation Attenuates Glycated-Serum Induced Pancreatic Beta-Cell Dysfunction through Enhancing Pdx1 and Mafa Protein Stability

**DOI:** 10.1371/journal.pone.0056386

**Published:** 2013-02-12

**Authors:** Yunxia Zhu, Ai Ma, Hongxiu Zhang, Chaojun Li

**Affiliations:** 1 The Jiangsu Key Laboratory for Molecular and Medical Biotechnology, College of Life Sciences, Nanjing Normal University, Nanjing, China; 2 MOE Key Laboratory of Model Animals for Disease Study, Model Animal Research Center and the Medical School of Nanjing University, National Resource Center for Mutant Mice, Nanjing, China; 3 Department of Obstetrics and Gynecology, First Affiliated Hospital of Nanjing Medical University, Nanjing, China; University of Texas Health Science Center at Houston, United States of America

## Abstract

Pancreatic-duodenal homeobox-1 (Pdx1) and v-maf musculoaponeurotic fibrosarcoma oncogene homolog A (Mafa) play important roles in sustaining the pancreatic beta-cell differentiation phenotype. Peroxisome proliferator-activated receptor-γ (PPARγ) is also a regulator of cell differentiation. Our previous study revealed that glycated serum (GS) causes beta-cell dedifferentiation by down-regulating beta-cell specific genes, such as *insulin* and *Pdx1*. Here, we show that GS enhanced the cellular accumulation of ubiquitin-conjugated proteins, including Pdx1 and Mafa, in pancreatic beta-cells. Pharmacologic inhibition of proteolytic activity restored the protein levels of Pdx1 and Mafa, whereas inhibition of *de novo* protein synthesis accelerated their degradation. These findings suggest that both Pdx1 and Mafa are regulated at the post-transcriptional level. We further show that activation of PPARγ could restore GS-induced reduction of Pdx1 and Mafa protein levels, leading to improved insulin secretion and synthesis. Moreover, ectopic expression of Bcl-xl, a mitochondrial regulator, also restored Pdx1 and Mafa protein levels, linking mitochondrial function to Pdx1 and Mafa stability. Taken together, our results identify a key role of PPARγ in regulating pancreatic beta-cell function by improving the stability of Pdx1 and Mafa proteins.

## Introduction

Advanced glycation end products (AGEs) are formed by nonenzymatic glycation and oxidation of proteins, lipids and nucleic acids, normally during aging, inflammation, renal failure and diabetes [1], [2]. Hyperglycemia in the diabetic setting accelerates the generation of AGEs [3]. Irreversible changes of diabetes, such as nephropathy [4], neuropathy [5] and atherosclerosis [6] are highly associated with accumulation of AGEs. Recently, several studies have revealed that the pancreatic islet beta-cell is also a target of AGEs [7], [8]. AGEs contribute to the deterioration in beta-cell function by inhibition of *insulin* gene transcription, degranulation of beta-cells and eventually abatement in beta-cell mass [9], [10], [11].

Pdx1, Mafa and Neurod1 are transcription factors that directly bind to the *insulin* gene promotor and serve as key regulators in pancreatic beta-cell differentiation and mature beta-cell function. A large body of evidence has shown that decreased nuclear levels of Pdx1, Mafa or Neurod1 lead to dedifferentiation of beta-cells and consequently inadequate insulin secretion in diabetes [12], [13], [14]. Moreover, numerous studies have demonstrated that simultaneous expression of Pdx1, Mafa and Neurod1 strikingly induces transdifferentiation of non-beta-cells, such as liver cells, to insulin-producing cells, thereby becoming very useful surrogates for beta-cells [15], [16]. Glucotoxicity, lipotoxicity and cytotoxic cytokines are well-known factors for progressive loss of beta-cell function and mass, and regardless of which signaling pathway is considered, compromising protein levels of Pdx1, Mafa or Neurod1 are involved to some extent [12], [13], [14]. However, knowledge of how to rescue those protein levels and maintain beta-cell differentiation status under the diabetic setting is still limited. Our previous study indicated that the effects of AGEs are more harmful than glucotoxicity in the development and progression of diabetes. AGEs compromise beta-cell function through the AGE-RAGE (receptor for AGE) pathway, and the effects can be attributed mainly to Pdx1 protein reduction [9]. Therefore, reviving Pdx1 protein levels may be a feasible way to maintain normal pancreatic beta-cell function in the presence of AGEs.

Peroxisome proliferator-activated receptor-γ (PPARγ) is a member of the nuclear hormone receptor superfamily of ligand-gated transcription factors. PPARγheterodimerizes with the retinoid X receptor (RXR) and binds to specific peroxisome proliferator hormone response elements (PPREs) on the DNA of target genes [17]. Thiazolidinediones (TZDs), such as troglitazone (TRO), are synthetic PPARγ agonists that influence diverse biological functions, including cellular differentiation, pro-survival and anti-proliferative processes, and glucose and lipid homeostasis [18]. Approved for the treatment of type 2 diabetes, PPARγ agonists can improve glucose disorders mainly through insulin-sensitizing effects in muscle and adipose tissue [19]. However, since PPARγis expressed in beta-cells in both rodents and humans [20], [21], the therapeutic effect of TZDs may be also mediated directly through the pancreatic islets. Treatment of diabetic or prediabetic humans and rodents with PPARγ agonists has been shown to contribute to improvements in islet architecture, insulin biosynthesis and glucose-stimulated insulin secretion (GSIS), and TRO was found to directly improve GSIS in isolated islets from fatty Zucker rats as well [22], [23]. Additionally, PPRE sequences have been reported on the promoters of *glucose transporter 2 (Slc2a2)* [24], *glucokinase (Gck)* [25] and *Pdx1* [26], [27]. Up-regulating the expression of those genes induced by PPARγ agonists helps to increase glucose sensitivity, insulin synthesis and GSIS in cultured primary rat islets and beta-cell lines [24], [25], [26], [27]. Our previous studies investigating the molecular basis of the toxicity of AGEs demonstrated that it results from the deficiency of mitochondrial function by inhibiting *Bcl2* and *Bcl2l1* expression, as well as decreased stability and level of the Pdx1 protein [9], [11]. Based on the known protective activity of TZDs drugs, we speculated whether the activation of PPARγ would counter the harmful effects of AGEs in pancreatic beta-cells and reverse or prevent the damage to the insulin-producing phenotype.

In the present study, primary rat islets and INS-1 cells were used to observe the effect of a PPARγ agonist, TRO, on glycated serum (GS) induced changes, and the underlying mechanism of this drug in maintaining the mature state of pancreatic beta-cells was explored.

## Materials and Methods

### AGE-fetal bovine serum (FBS) preparation

GS was prepared as described previously [9]. FBS (Hyclone) was incubated under sterile conditions with D-glucose (90 g/L) at 37°C for 3 weeks. Unincorporated sugars were then removed by dialysis against 0.2 mol/l phosphate-buffered saline (PBS). Control nonglycated serum (NG) was incubated under the same conditions but without D-glucose. Application of the Limulus amebocyte assay before the *in vitro* study revealed that the reagents contained less than 0.2 ng/mL of endotoxin.

### Cell culture

Rat insulinoma INS-1 cells [28] between passages 18–32 were grown in RPMI-1640 medium (Invitrogen) containing 11.1 mM D-glucose, 1 mM sodium pyruvate, 10% FBS, 100 U/mL penicillin, 100 mg/mL streptomycin, 10 mM HEPES, 2 mM L-glutamine and 50 mM β-mercaptoethanol (Sigma-Aldrich). Cells were maintained at 37°C in an atmosphere of 95% O_2_/5% CO_2_. Before the addition of GS, the cells were gently washed in PBS. NG or GS was added to the appropriate cell cultures and incubated for the indicated time(s). GS was used at the concentration of 10% in this study unless otherwise specified.

### Pancreatic islet isolation

All animal studies were performed according to guidelines established by the Research Animal Care Committee of Nanjing Medical University. Male Sprague–Dawley rats (200–250 g) were purchased from Shanghai Laboratory Animal Centre (Chinese Academy of Sciences, China). Islet isolation and culturing techniques have been described previously [11]. Freshly isolated islets were transferred to sterile 6-cm dishes and cultured in RPMI 1640 containing 11.1 mmol/l glucose supplemented with 10% FBS, 10 mmol/l HEPES, 100 U/ml penicillin and 100 lg/ml streptomycin. The islets were allowed to equilibrate for 3 h, after which they were counted, transferred to a 48-well plate (10 islets/well) and cultured overnight at 37°C. The following morning, the islets were pre-treated with TRO for 1 h and then cultured with NG, 2% GS or 5% GS in the depletion medium. GSIS studies were performed 48 h later.

### Plasmid construction and transient transfections

The 3×PPRE-TK-Luc reporter construct was made as previously described [29]. pBK-CMV-hPPARγ and pORF-hBcl-xL plasmids were purchased from Invitrogen. The rat Pdx1 expression plasmid was constructed by inserting the full-length coding region sequence into pCMV5 vector between XhoI and HindIII, and the mouse Mafa expression plasmid was constructed by inserting the full-length coding region sequence into pAdTrack-CMV vector between HindIII and BamHI. All constructions used here were sequenced and confirmed to be correct. Transient transfections were carried out by using Lipofectamine 2000 transfection reagent (Invitrogen) following the instructions. For the luciferase reporter assay, INS-1 cells were seeded in 24-well plates, and the 3×PPRE-TK-Luc plasmid (0.4 µg) was co-transfected with pBK-CMV-hPPARγ(0.4 µg) or vector control. Twenty-four hours post-transfection, cells were treated with NG or GS with or without 20 µmol/l TRO for an additional 16 h. Luciferase activities were measured using a dual-luciferase reporter assay system (Promega). The firefly luciferase activity was normalized with the *Renilla* activity of the pRL-SV40 vector (0.005 µg) (Promega). For immunoblotting analysis, INS-1 cells were seeded in 35-mm dishes, and PPARγ (4 µg) or Bcl-xL (4 µg) were transfected for 24 h, followed by NG or GS treatment for another 16 h before whole protein extraction. 293A cells or INS-1 cells were seeded in 60-mm dishes, and indicated plasmids (6 µg) were transfected into cells for 36 h, at which time cells were extracted for coimmunoprecipitation (co-IP) assay.

### Real-time RT-PCR

INS-1 cells (2×10^6^ cells per well) were seeded in 35-mm dishes and treated with NG or GS as described above. Total RNA was extracted using Trizol reagent according to the manufacturer's protocol (Invitrogen). First-strand cDNA was synthesized from 1 µg of total RNA in 20 µl total volume using an oligo-dT primer and an avian myeloblastosis virus reverse transcription system (Promega). Specific primers were designed using the software Primer Express (Applied Biosystems). The sequences of the primers used are available in Table S1. Quantitative real-time PCR was performed using the SYBR Green PCR Master Mix and Roche LightCycle480 II Sequence Detection System (Roche Diagnostics). *β-Actin* was used as an internal control for quality and quantity of RNA.

### Co-immunoprecipitation and Western blot analysis

INS-1 cells were cultured and treated as described above and then lysed with ice-cold lysis buffer A containing the following reagents: 50 mM Tris-HCl (pH 7.4), 1% NP-40, 150 mM NaCl, 1 mM EDTA, 1 mM phenylmethylsulfonyl fluoride and Complete proteinase inhibitor (one tablet per 10 mL; Roche Molecular Biochemicals, Indianapolis, IN). After protein content determination, Western blotting was performed as described previously [9]. The boiled samples were separated on 10% SDS-polyacrylamide gels and transferred to Immun-Blot® PVDF membranes (Bio-Rad). After blocking with 5% skim milk in PBS with 0.1% Tween 20 for 1 h, the membranes were probed with the following antibodies: rabbit anti-PPARγ antibody (Santa Cruz Biotechnology), rabbit anti-Bcl-xl antibody (Santa Cruz Biotechnology), rabbit anti-Pdx-1 antibody (Upstate), rabbit anti-Mafa antibody (Santa Cruz Biotechnology), mouse anti-β-Actin antibody (Sigma-Aldrich) and mouse anti-β-Tubulin antibody (Sigma-Aldrich). Bound antibodies were visualized by enhanced chemiluminescence (Amersham Pharmacia Biotech) using horseradish peroxidase-conjugated antibodies (Millipore).

For co-immunoprecipitation, transiently transfected 293A or INS-1 cells in 60-mm dishes were lysed with lysis buffer B (20 mM Tris-HCl, pH 7.4, 150 mM NaCl, 1 mM EDTA, 1 mM EGTA, 1% Triton, 2.5 mM sodium pyrophosphate, 1 mM β-glycerolphosphate, 1 mM sodium orthovanadate, 1 mg/ml leupeptin, 1 mM phenylmethylsulfonyl fluoride), sonicated three times for 5 s each and then centrifuged at 13,000 rpm for 20 min at 4°C. Flag-tagged ubiquitin, Pdx1 or Mafa proteins were immunoprecipitated from the cell lysate with an anti-Flag, anti-Pdx1 or anti-Mafa antibody, respectively, and Protein A/G Plus agarose beads. Immunoprecipitates or total cell lysates were analyzed by Western blotting as described above. Individual immunoblots were probed with either mouse anti-Flag antibody (Sigma-Aldrich), rabbit anti-ubiquitin antibody (Santa Cruz Biotechnology), rabbit anti-Pdx-1 antibody (Upstate), rabbit anti-Mafa antibody (Santa Cruz Biotechnology) or mouse anti-β-Tubulin antibody (Sigma-Aldrich).

### Insulin secretion and insulin content

Islets were transferred to 48-well plates (10 islets/well). INS-1 cells also were seeded in 48-well plates. After 24 h, cells were pre-cultured with DMSO or 20 µmol/l TRO for 1 h, followed by treatment with NG or GS for an additional 48 h for islets or 24 h for INS-1 cells. After washing twice with PBS (pH 7.4), islets or INS-1 cells were pre-incubated in HEPES-balanced Krebs-Ringer bicarbonate buffer (KRBH) containing 3.3 mmol/l glucose and 1 g/L bovine serum albumin (BSA) for 1 h. Then, cells were incubated for 1 h in KRBH containing 3.3 mmol/l glucose (basal), followed by stimulation for 1 h in KRBH containing 16.7 mmol/l glucose or 50 mmol/l potassium. After the static incubation, supernatants were collected and frozen at −80°C. To determine insulin content, cells were washed twice with PBS (pH 7.4) at 0°C and extracted with acid/ethanol (0.15 M HCl in 75% ethanol in H_2_O) for 16 h at 0°C. Supernatants were collected and stored at −80°C for subsequent determination of the insulin concentration. Insulin secretion and content were performed using a radioimmunoassaay (RIA) kit as described previously. The glucose-stimulated insulin secretion index (GSI) and the potassium-stimulated insulin secretion index (KSI) were each calculated as the ratio of stimulated-insulin release to basal release. Insulin content was normalized to the total protein concentration.

### Statistical analysis

Data are presented as means ± SEM. Differences between groups were compared by 2-tailed Student's *t* test. A *P* value of less than 0.05 was considered to be statistically significant.

## Results

### GS inhibits insulin synthesis and secretion

**Figure 1:**
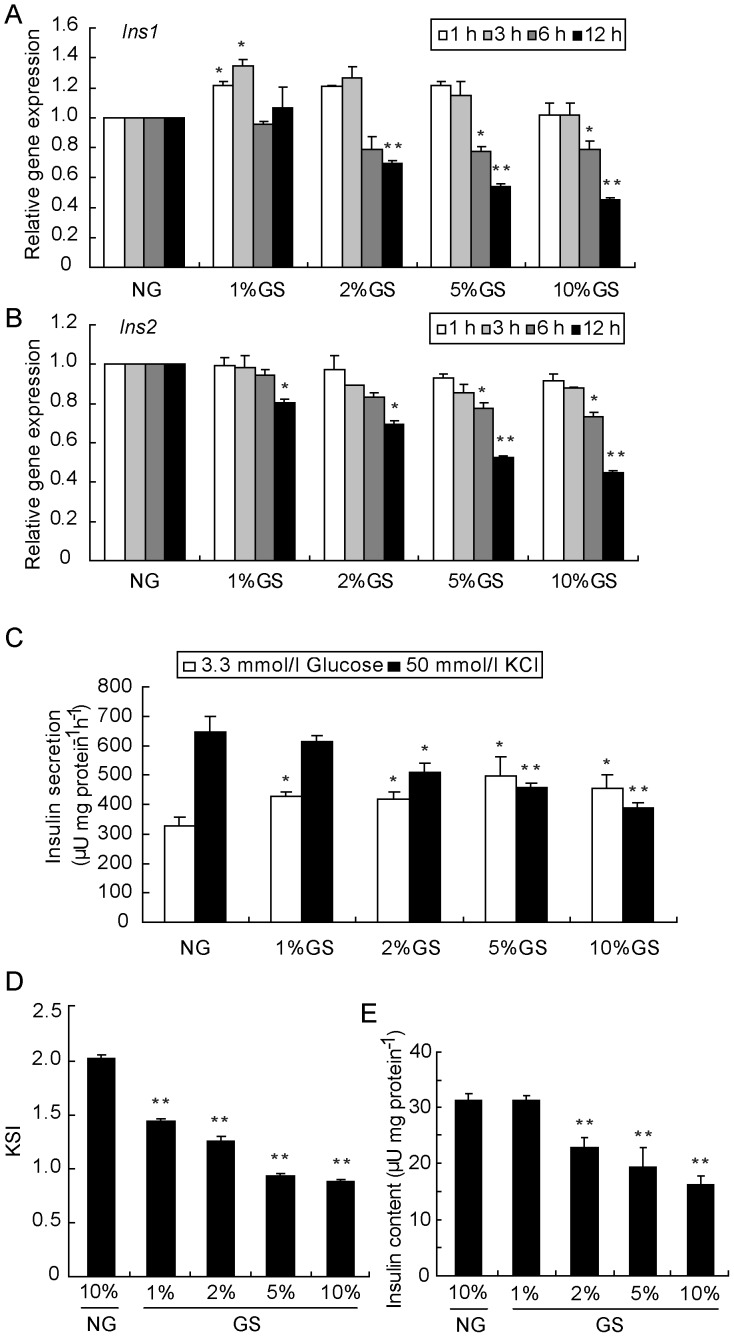
GS inhibits insulin synthesis and secretion in INS-1 cells. INS-1 cells were treated with NG, 1% GS, 2% GS, 5% GS or 10% GS for 1, 3, 6 and 12 h, and then *Ins1* (A) and *Ins2* (B) gene expression levels were analyzed by real-time RT-PCR. After treatment of INS-1 cells with the indicated concentration of GS for 24 h, the KSIS assay (C) was carried out, and the potassium-stimulated insulin secretion index (KSI) (D) and total insulin in the cellular extract (E) were determined. The insulin level was determined by RIA. Insulin secretion and content were normalized by the total protein concentration. Values are the mean ± SEM of three individual experiments. *P<0.05 vs. NG; **P<0.01 vs. NG.

Insulin secreted mainly by pancreatic islet beta-cells is the only hormone that reduces the plasma glucose level. Relatively or absolutely inadequate secretion of insulin leads to the development of diabetes. Chronic hyperglycemia is thought to accelerate formation of AGEs. To simulate the chronic diabetic setting, we prepared the GS by incubating a mixture of D-glucose and FBS for a 3-week period. GS contains heterogeneous AGEs and may mimic the diabetic microenvironment *in vivo*. To test the harmful effect of AGEs, mRNA levels of *Ins1* and *Ins2* were analyzed by real-time RT-PCR over time in INS-1 cells after treatment with GS at various concentrations. Although there was a slight increase, the reduction of *Ins1* was initiated after 3 h of treatment with 5% GS and 6 h of treatment with 2% GS (Fig. 1A). On the other hand, the *Ins2* level was down-regulated by GS in a time- and dose-dependent manner (Fig. 1B). Due to the lack of glucose response in INS-1 cells, we performed the potassium-stimulated-insulin secretion (KSIS) assay to evaluate insulin secretion ability. As expected, insulin secretion was reppressed by stimulation with 50 mmol/l KCl in cells treated with 2–10% GS for 16 h (Fig. 1C). Moreover, due to the increase in the basal level of insulin secretion, the insulin secretion index was decreased even at the 1% GS dosage (Fig. 1D). To determine whether the observed changes in the mRNA level of *insulin* genes would correspond to defective insulin production, the insulin content was detected after treatment of INS-1 cells with different concentrations of GS for 24 h. Consistent with the alteration in insulin mRNAs, the protein level was also significantly reduced by GS starting at the dosage of 2% (Fig. 1E).

### Instability of Pdx1 and Mafa protein production in INS-1 cells by treatment with GS

**Figure 2:**
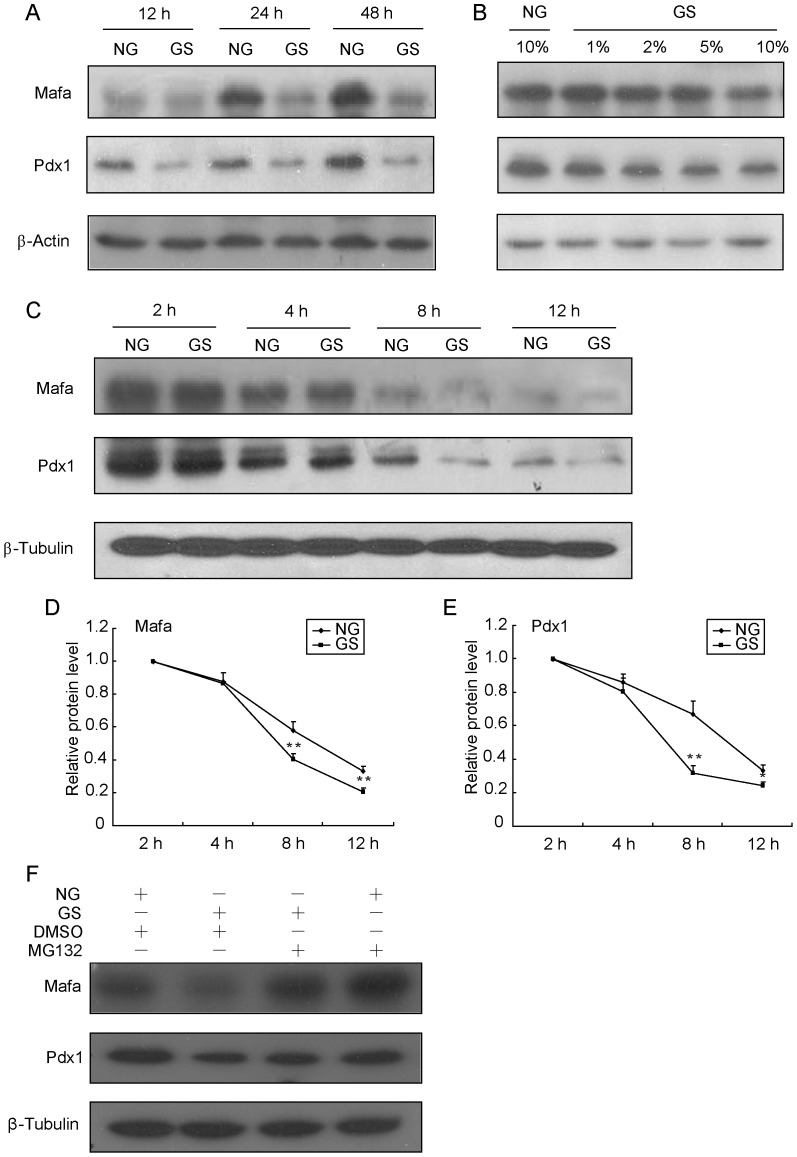
GS induces instability of Pdx1 and Mafa proteins. INS-1 cells were treated with 10% GS for the indicated times (A) or with different GS concentrations for 16 h (B), and the protein levels of Mafa and Pdx1 were determined by Western blot analysis. (C) INS-1 cells were pre-treated with 50 µmol/l cycloheximide for 2 h and then co-treated with NG or 10% GS for 2, 4, 8 or 12 h. Total proteins were extracted and analyzed by Western blot analysis. (D) Pdx1 and Mafa protein levels normalized to β-Tubulin were compared with the 2 h protein level. (F) INS-1 cells were treated with NG and 10% GS for 12 h, and then MG132 or DMSO was added for an additional 4 h before proteins were harvested to perform Western blot analysis. Values are the mean ± SEM of three individual experiments. *P<0.05 vs. NG; **P<0.01 vs. NG.

Three transcriptional factors, Pdx1, Mafa and Neurod1, equally share functions as beta-cell specific regulators. To identify which transcriptional regulator(s) contribute to GS-induced impairment of insulin synthesis, we monitored the protein levels of Pdx1, Mafa and Neurod1 by immunoblotting of INS-1 cells. Pdx1 and Mafa protein levels were decreased significantly after treatment with 10%GS for 12 h and thereafter persistently decreased over time (Fig. 2A). The levels of these two proteins were also decreased by treatment with GS over a range of different concentrations (1–10%) for 16 h (Fig. 2B). However, Neurod1 and Hnf1a protein levels were not affected (data not shown). Considering no significant change in gene expression of *Mafa* was observed within 12 h, while the mRNA and protein levels of Pdx1 were altered almost simultaneously from 4 h (data not shown), we speculated that GS may affect Pdx1 and Mafa proteins mainly at the post-transcriptional level. To test this hypothesis, we determined the stability of these proteins in INS-1 cells treated with NG or GS for 2, 4, 8 or 12 h in the presence of 50 µmol/l cycloheximide. After *de novo* protein synthesis was blocked by cycloheximide, GS caused a more rapid reduction in Pdx1 and Mafa protein levels compared with NG treatment (Fig. 2C, D, E). Next, we treated cells with NG and GS for 12 h and added MG132 or DMSO to the culture for an additional 4 h before cells were harvested to perform Western blotting. MG132 treatment significantly stabilized Pdx1 and Mafa protein levels (Fig. 2F). As a specific proteasome inhibitor, MG132 reduces the degradation of ubiquitin-conjugated proteins in mammalian cells. These results suggested that GS regulates Pdx1 and Mafa expression at the post-translational level by increasing their degradation through the ubiquitin-proteasome proteolytic pathway.

### Ubiquitin modification contributes to the instability of Pdx1 and Mafa

**Figure 3:**
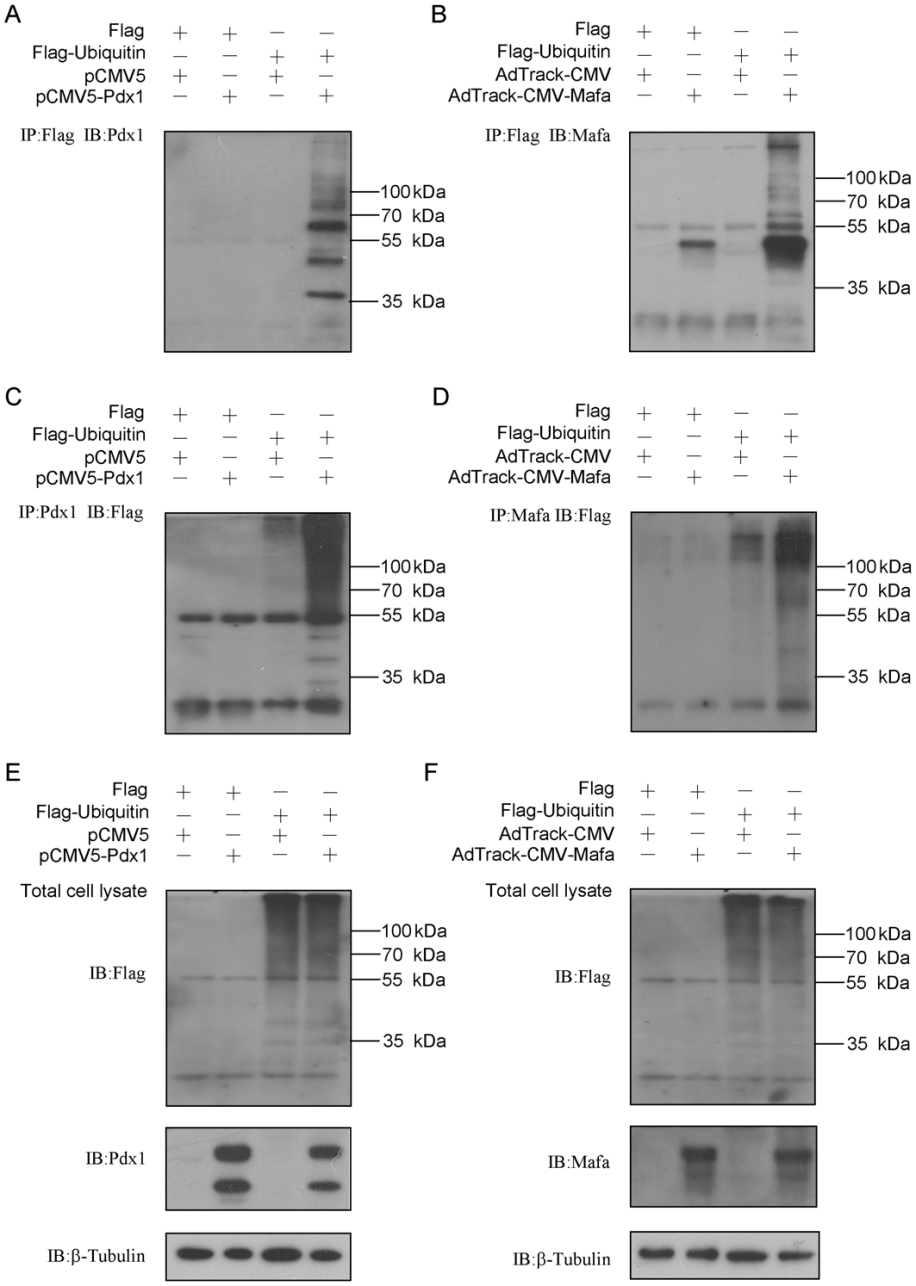
Pdx1 and Mafa are targets of ubiquitin modification. (A, B) Flag-ubiquitin was co-expressed in 293A cells with or without the Pdx1 plasmid for 36 h. The cells were then subjected to co-immunoprecipitation with an anti-Flag or anti-Pdx1 antibody, and the products were immunoblotted to detect Pdx1 (A) or Flag (B). (C, D) Flag-ubiquitin was co-expressed in 293A cells with or without the Mafa plasmid for 36 h. The cells were then subjected to co-immunoprecipitation with an anti-Flag or anti-Mafa antibody, and the products were immunoblotted to detect Mafa (C) or Flag (D). (E, F) 293A cells were transfected with indicated plasmids as above for 36 h and then analyzed by immunoblotting.

Next, to investigate whether the ubiquitin protein could modify Pdx1 and Mafa, we performed an *in vivo* ubiquitination assay in 293A cells. Flag-tagged ubiquitin was co-expressed with or without Pdx1 or Mafa for 36 h in 293A cells. Cells were subjected to immunoprecipitation with an anti-Flag antibody, and the products were immunoblotted for Pdx1 or Mafa. Ubiquitination of Pdx1 or Mafa was detectable as a ladder of high-molecular-weight bands (Fig. 3A, B). On the other hand, when cells were subjected to immunoprecipitation with either Pdx1 or Mafa, Flag-ubiquitin was also co-precipitated in a high-molecular-weight form (Fig. 3C, D). Moreover, the protein levels of Pdx1 and Mafa were significantly down-regulated when they were co-expressed with ubiquitin (Fig. 3E, F). These results indicated that ubiquitin could modify Pdx1 and Mafa and eventually led to their decreased stability.

### Synergistic effects of GS and TRO in stimulating PPARγ activation

**Figure 4:**
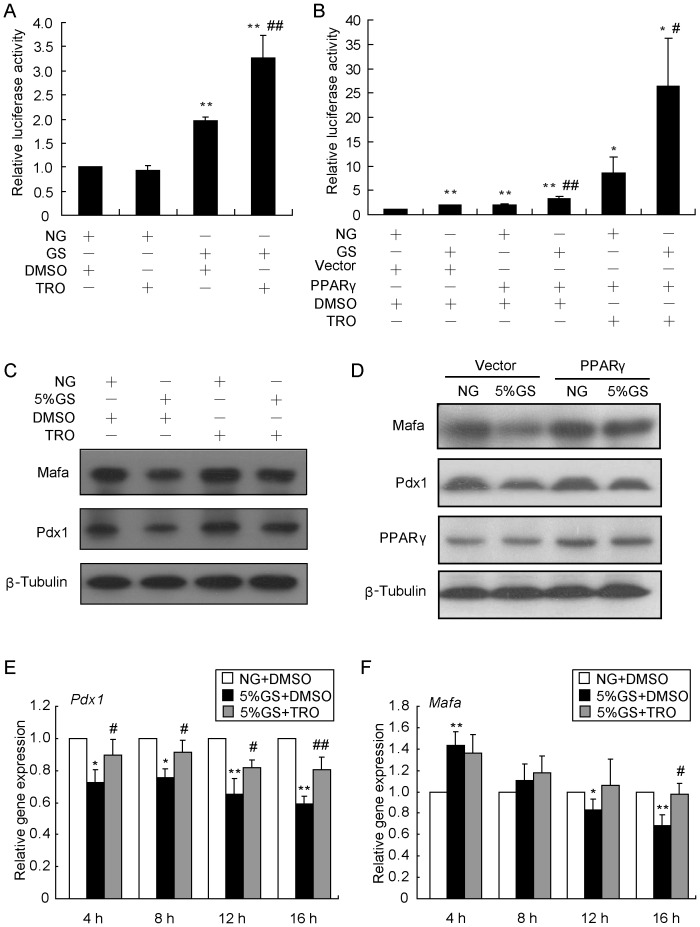
TRO normalizes Pdx1 and Mafa protein levels. (A) INS-1 cells were transfected with the PPRE-luc plasmid for 24 h and then treated with NG or 10% GS for an additional 16 h before analysis in a dual-luciferase reporter assay. The firefly luciferase activity representing PPARγ activity was normalized to the *Renilla* activity of pRL-SV40. **P<0.01 vs. NG + DMSO; ^# #^ *P*<0.01 vs. GS + DMSO. (B) After co-transfection with PPRE-luc and PPARγor vector for 24 h, INS-1 cells were treated with NG or 10% GS for another 16 h. Cells extracts were analyzed using the dual-luciferase reporter assay. *P<0.05 or **P<0.01 vs. NG + vector + DMSO; ^#^ *P*<0.05 or ^# #^ *P*<0.01 vs. NG. (C) INS-1 cells were pre-treated with DMSO or 20 µmol/l TRO for 1 h and then exposed to NG or 5% GS for 16 h before detection of Pdx1 and Mafa levels by Western blot. β-Tubulin was used as an internal standard. (D) After transfection with the PPARγ over-expression plasmid for 24 h, INS-1 cells were treated with NG or 5% GS for an additional 16 h before harvesting for Western blot analysis of Pdx1 and Mafa levels. (E, F) INS-1 cells were pre-cultured with 20 µmol/l TRO for 1 h and then co-cultured with NG and 5% GS for the indicated time before analysis of *Pdx1* (E) and *Mafa* (F) mRNA levels by real-time RT-PCR. *β-Actin* was used as an internal standard. Values are the mean ± SEM of three individual experiments. *P<0.05 or **P<0.01 vs. NG; ^#^ *P*<0.05 or ^# #^ *P*<0.05 vs. 5% GS.

Given the beneficial effects of PPARγ agonists in type 2 diabetes, their molecular mechanisms of decreasing blood glucose have been widely studied. Among them, TRO has become a useful tool for investigating the function of PPARγ activation. Here, the transcriptional activity of PPARγ was evaluated by using a PPRE luciferase reporter assay. We found that GS treatment led to activation of PPARγ and the effect was enhanced by TRO administration (Fig. 4A). More surprisingly, over-expression of PPARγ was capable of increasing the GS-induced activation of PPARγ with or without TRO administration(Fig. 4B). The results demonstrated that GS and TRO had a positive synergistic effect in activating PPARγ.

### Activation of PPARγ rescues Pdx1 and Mafa protein synthesis

Since GS significantly stimulated PPARγ activation, we wanted to investigate the consequence of this phenomenon. Therefore, we tested the effect of 20 µmol/l TRO pre-treatment for 1 h on protein levels of Pdx1 and Mafa. In INS-1 cells treated with GS for 16 h, Pdx1 and Mafa were significantly reduced, while TRO pre-treatment rescued these protein levels dramatically (Fig. 4C). To confirm these results, we transfected a PPARγ expression construct into INS-1 cells for 24 h, and then cultured the cells with NG or GS for another 16 h. Over-expression of PPARγ resulted in partial recovery of Pdx1 and Mafa protein levels as well (Fig. 4D). These results suggested that activated PPARγ could maintain normal Pdx1 and Mafa protein levels. Because Pdx1 is a reported transcriptional target of PPARγ, the mRNA levels of *Pdx1* and *Mafa* may also contribute to recovery of their proteins. Fig. 4E shows that *Pdx1* gene expression was down-regulated with the addition of GS for different times, from 4 h to 16 h, and the down-regulation was slightly inhibited with TRO pre-treatment. Meanwhile, the change of *Mafa* expression was analyzed in the same condition. With a slight increase at 4 h, *Mafa* expression was inhibited after GS treatment for 12 h. Thus, the effect of TRO in regulating *Mafa* gene expression appeared to be moderate (Fig. 4F).

### PPARγ activation post-translationally regulates Pdx1 and Mafa protein levels

**Figure 5:**
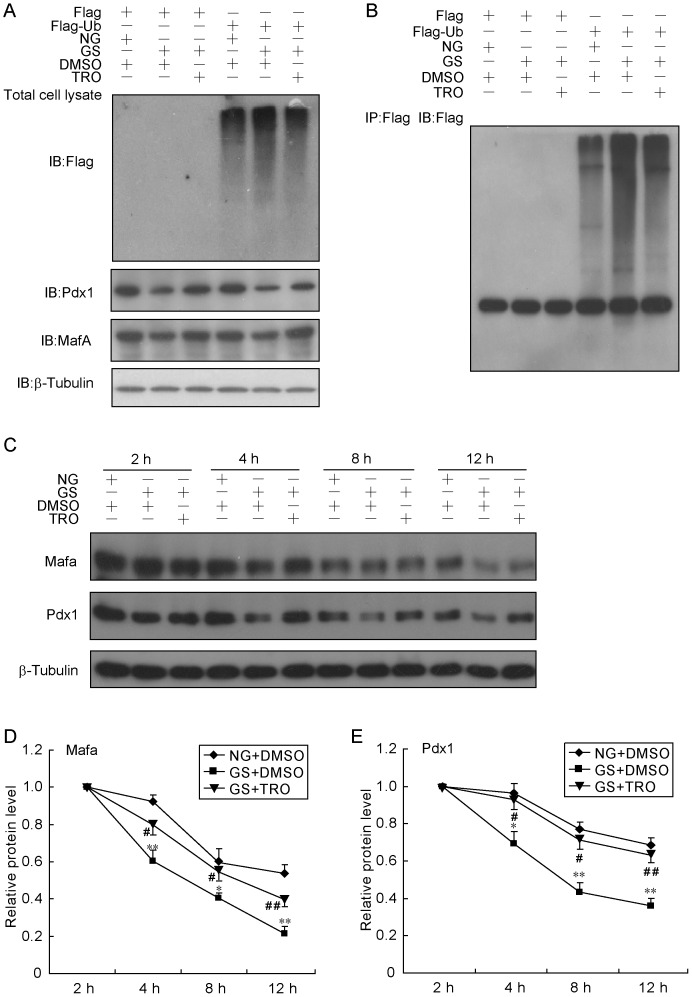
TRO post-translationally regulates Pdx1 and Mafa. (A, B) After transfection with Flag or Flag-ubiquitin for 24 h, INS-1 cells were treated with GS with or without 20 µmol/l TRO for 16 h before harvesting. Ten percent of the total cell lysate was used to measure protein expression by immunoblotting (A). The rest of the total cell lysate was subjected to co-immunoprecipitation with anti-Flag, and the product was analyzed by immunoblotting (B). (C, D, E) INS-1 cells were divided into three groups (NG+DMSO, GS+DMSO and GS+20 µmol/l TRO). After the indicated treatments for 2 h, cells were co-cultured with 50 µmol/l cycloheximide for 2, 4, 8 or 12 h. Total proteins were extracted and analyzed using immunoblotting (C). The half-lives of Pdx1 (D) and Mafa (E) were calculated.

Based on the above results, it seemed difficult to determine whether Pdx1 and Mafa protein levels are regulated at the transcriptional or post-transcriptional level. To clarify the function of PPARγ activation, we first tested its effect on protein ubiquitination. Transfection of Flag-ubiquitin into INS-1 cells caused the accumulation of high-molecular-weight ubiquitinated proteins, and the effect was enhanced by GS treatment in either whole cell lysate (Fig. 5A) or immunoprecipitation products (Fig. 5B). Moreover, TRO pre-treatment of cells inhibited the accumulation of GS-induced ubiquitination of proteins. Meanwhile, when cells were pre-cultured with TRO, the compromised protein production of Pdx1 and Mafa by GS treatment recovered to almost normal levels in INS-1 cells transfected with either a Flag-ubiquitin or Flag vector plasmid (Fig. 5A). Next, by using cycloheximide to block *de novo* protein synthesis, we measured the half-lives of Pdx1 and Mafa. Like the previous result, GS induced a rapid protein degradation rate and a short half-life for both Pdx1 and Mafa, while TRO pre-treatment prevented these effects (Fig. 5C, D, E). Our data suggested that TRO regulates Pdx1 and Mafa protein levels at the post-translational level.

### Activation of PPARγ rescues insulin expression and beta-cell KSIS function

**Figure 6:**
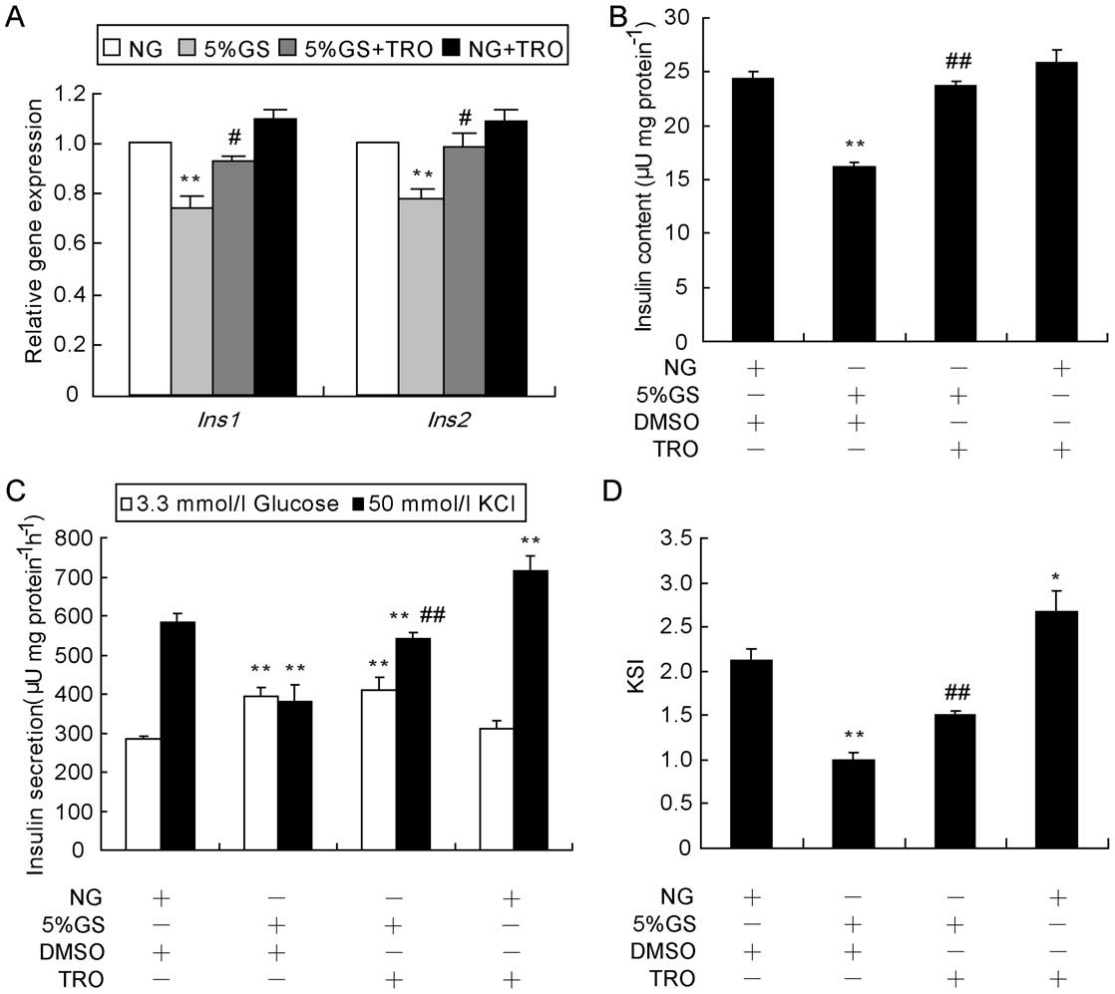
Activation of PPARγ rescues *insulin* gene expression and insulin secretion. (A) Cells were pre-treated with TRO or DMSO for 1 h, followed by treatment with NG or 5% GS for 8 h, and then total RNA was extracted using Trizol reagent. Real-time RT-PCR was utilized to determine mRNA levels of *Ins1* and *Ins2*. *β-Actin* was used as an internal standard. **P<0.01 vs. NG; ^#^ *P*<0.05 or ^# #^ *P*<0.01 vs. 5% GS. INS-1 cells were treated with NG or 5% GS with or without 20 µmol/l TRO for 24 h. Thereafter, the KSIS assay (C) was carried out, and the potassium-stimulated insulin secretion index (KSI) (D) and total insulin in the extract (B) were determined. The insulin level was determined by RIA. Insulin secretion and content were normalized by total protein concentration. *P<0.05 and **P<0.01 vs. NG + DMSO; ^# #^ *P*<0.01 vs. 5% GS + DMSO.

To determine whether the recovery of Pdx1 and Mafa protein levels by activation of PPARγ would contribute to insulin secretion and synthesis, we first analyzed the mRNA levels of *insulin* genes. Expression levels of both *Ins1* and *Ins2* genes were down-regulated by GS after 8 h, and they were restored when cells were pre-treated with TRO for 1 h (Fig. 6A). We then performed the KSIS assay to analyze insulin secretion. Cells were extracted with acid/ethanol to determine total insulin content, which was found to be almost completely recovered (Fig. 6B). The KSIS index was partially rescued, mainly due to an increased potassium-stimulated insulin level and not to decreased basic insulin secretion (Fig. 6C, D). Moreover, TRO also benefited KSIS in the NG treated group (Fig. 6C). Our data suggested that the recovery of insulin synthesis and secretion from GS treatment might be associated with the normalization of Pdx1 and Mafa proteins resulting from PPARγ activation.

### Mitochondrial impairment, not endoplasmic reticulum (ER) stress or glucose transport abnormality, is involved in GS-induced beta-cell dysfunction

**Figure 7:**
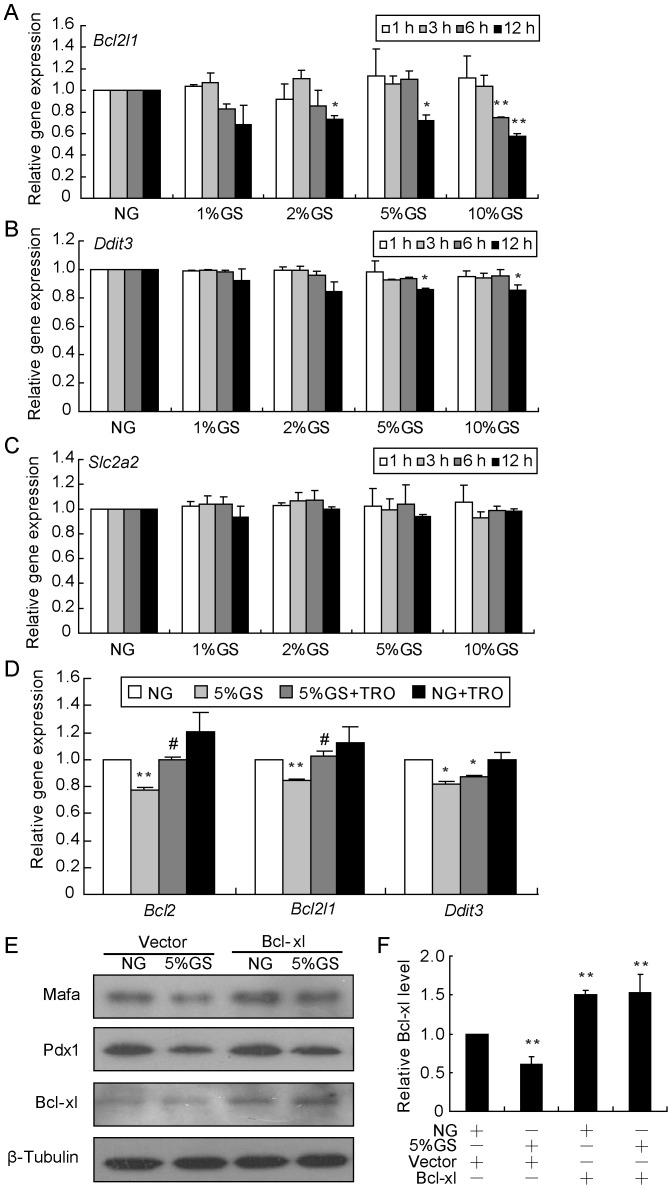
Mitochondrial dysfunction, not ER stress or glucose transport abnormality, is involved in GS-induced damage. INS-1 cells were treated for the indicated time with GS ranging in the concentration from 1% to 10%, and then *Bcl2l1* (A), *Ddit3* (B) and *Slc2a2* (C) gene expression levels were analyzed by real-time RT-PCR. (D) INS-1 cells were pre-cultured with TRO or DMSO for 1 h followed by treatment with NG or 5% GS for 8 h, and then gene expression levels were determined by real-time RT-PCR. *P<0.05 and **P<0.01 vs. NG; ^#^ *P*<0.05 vs. 5% GS. (E) INS-1 cells were transfected with the Bcl-xl plasmid for 24 h, followed by NG or 5%GS treatment for 16 h. Protein levels of Mafa, Pdx1 and Bcl-xl were detected, with β-Tubulin was used as an internal standard.

In our previous study, we observed that GS caused morphological changes in the mitochondria and cytochrome c release from mitochondria to cytoplasm, accompanied by decreased *Bcl2* and *Bcl2l1* expression [11]. Here, we analyzed the mRNA levels of *Bcl2l1*, *Ddit3* and *Slc2a2* to monitor the impairment of mitochondria, ER, and glucose transport. As shown in Fig. 7A–C, in INS-1 cells cultured with GS, the *Bcl2l1* gene was reduced significantly in a time- and dose-dependent manner after normalization to *β-Actin*; however, little effect was found on *Ddit3* and *Slc2a2* expression. PPARγ agonists have been shown to successfully ameliorate mitochondrial function by increasing mitochondrial biogenesis and attenuating the mitochondrial membrane potential loss. Since *Bcl2* and *Bcl2l1* are recognized as functional genes for maintaining membrane potential, activation of PPARγ by TRO may facilitate the GS-induced mitochondrial dysfunction via a Bcl2 and Bcl-xl dependent manner. As we theorized, TRO was capable of preserving *Bcl2* and *Bcl2l1* but not *Ddit3* expression (Fig. 7D). Next, we wondered whether improving mitochondrial function by restoring Bcl2 or Bcl-xl protein levels would eliminate GS-induced damage. Indeed, transfection of INS-1 cells with a Bcl-xl overexpression construct normalized Pdx1 and Mafa synthesis (Fig. 7E).

### Activation of PPARγ rescues insulin gene expression and insulin release in primary rat pancreatic islets

**Figure 8:**
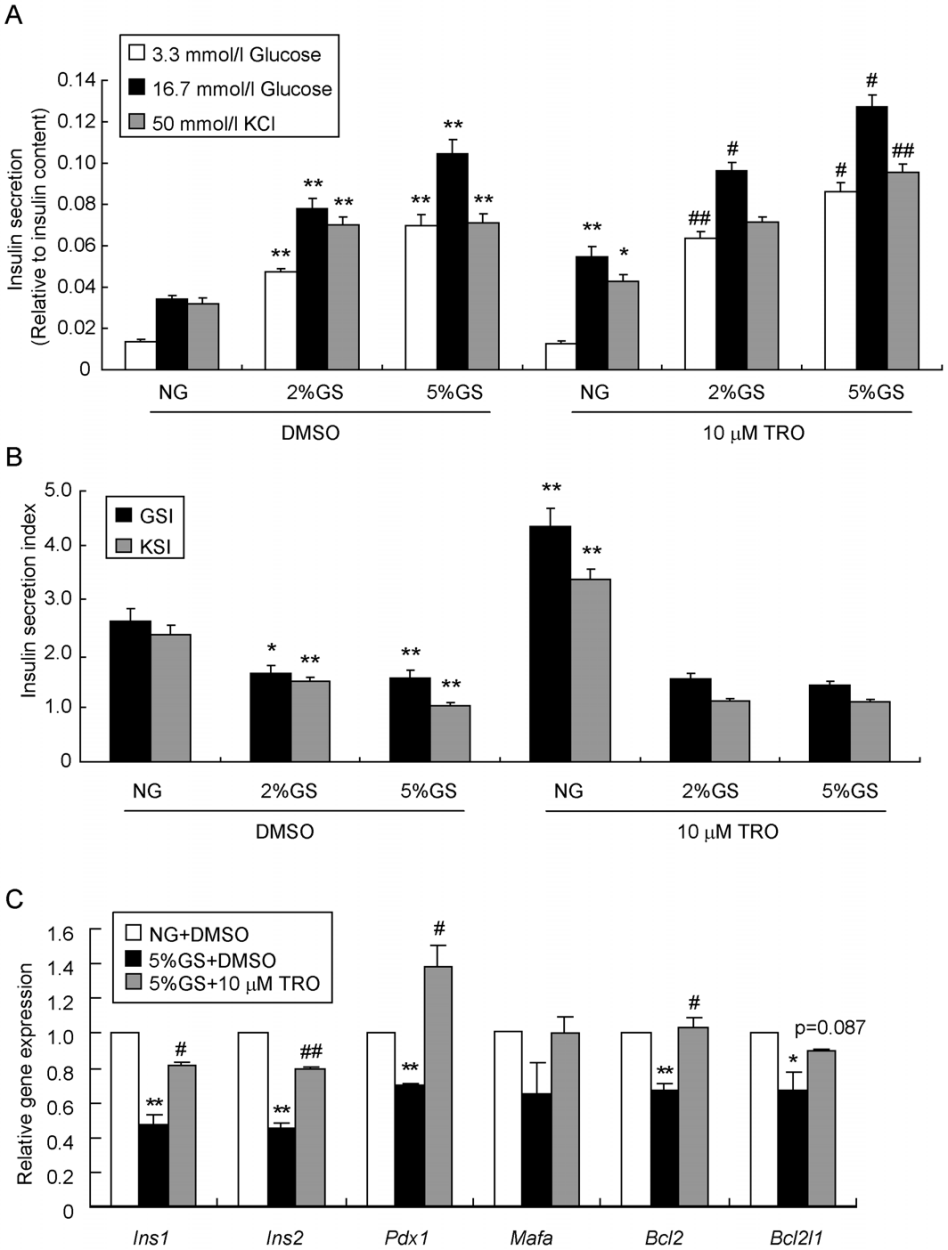
TRO restores *insulin* gene expression and insulin release in primary rat pancreatic islets. Islets isolated from SD rats were pre-cultured with or without 10 µmol/l TRO for 1 h, followed by treatment with NG, 2% GS or 5% GS for an additional 48 h. (A) GSIS and KSIS were determined. ^*^ *P*<0.05 or ^**^ *P*<0.01 vs. NG + DMSO; ^#^ *P*<0.05 or ^# #^ *P*<0.01 vs. 2% GS/5% GS + DMSO. (B) GSI and KSI were calculated as ratios of stimuli-induced insulin secretion relative to the basic low glucose level. ^*^ *P*<0.05 or ^**^ *P*<0.01 vs. NG + DMSO. (C) Real-time RT-PCR was carried out to determine the gene expression levels. ^*^ *P*<0.05 or ^**^ *P*<0.01 vs. NG + DMSO; ^#^ *P*<0.05 or ^# #^ *P*<0.01 vs. 5% GS + DMSO.

To confirm the results obtained from INS-1 cells, primary pancreatic islets isolated from Sprague-Dawley (SD) rats were tested. First, we investigated the effects of GS and TRO on insulin secretion in primary islets. GS ranging from 2% to 5% dose-dependently induced both basal levels and glucose- or potassium-stimulated levels of secreted insulin relative to the NG control (Fig. 8A), but the GSI and KSI were strikingly impaired (Fig. 8B). TRO only increased insulin secretion in response to high glucose or potassium. More surprisingly, TRO pre-treatment further enhanced the GS-induced insulin secretion (Fig. 8A), but no significant influence was observed on GSI and KSI (Fig. 8B). Second, we carried out real-time RT-PCR analysis to examine expression levels of relevant genes under these conditions. As shown in Fig. 8C, the mRNA levels of *Ins1*, *Ins2*, *Pdx1*, *Bcl2* and *Bcl2l1* were significantly down-regulated by 5% GS treatment, while no change was observed in *Mafa*; meanwhile TRO pre-treatment successfully rescued the expression of these genes. Overall, results from primary islets were similar to those obtained with INS-1 cells.

## Discussion

In the present study, we revealed that insulin synthesis and release was dramatically inhibited in a time- and dose-dependent manner by GS in INS-1 cells. Meanwhile, GS induced accumulation of ubiquitinated Pdx1 and Mafa proteins, accelerating their degradation rate and consequently reducing their intracellular levels. Surprisingly, cells pre-cultured with TRO or transfected with PPARγ resisted the damaging effects caused by GS. Based on our investigation, we speculated that activation of PPARγ normalizes the mitochondrial function and then restores Pdx1 and Mafa proteins, thereby ameliorating the defects in insulin secretion and synthesis induced by GS.

Chronic hyperglycemia in the diabetic setting accelerates the formation and accumulation of AGEs in the circulation [3]. AGEs have been found in many tissues including pancreatic islets. Much evidence has been found to show that AGEs are involved in diabetic complications, such as nephropathy [4], neuropathy [5], and atherosclerosis [6]. As pancreatic beta-cells is affected by diabetic complications [30], an increased level of blood AGEs likely contributes to failure of these cells in diabetic patients and rodents to some extent. To mimic the the chronic hyperglycemic condition in diabetic patients in this study, we prepared GS by mixing D-glucose and FBS at 37°C for 3 weeks instead of using AGEs. This preparation procedure was likely to include all possible components of AGEs and could mimic the complex serum components in diabetic patients. We and others have reported that AGEs dramatically induces pancreatic beta-cell impairment [7], [8], [9], [11], characterized by decreased insulin synthesis and secretion, as well as by cellular apoptosis. Although the particular mechanism is still not completely understood, all these studies suggest that mitochondrial dysfunction and oxidative stress might be associated with the induction of beta-cell impairment by AGEs. Oxidative stress results in an increase of misfolded proteins which are targeted by ubiquitin [31], [32], and the high-molecular-weight ubiquitinated proteins formed are selected for degradation. A previous study reported subcellular protein ubiquitination events during diabetes by visualizing pancreatic islets in a rat diabetic model [33]. *In vitro*, ubiquitinated protein aggregates were also found to be increased in INS-1 832/13 beta-cells cultured in high glucose [33]. Moreover, when the cells were removed from the high glucose environment and exposed to a basal glucose level, the ubiquitinated proteins aggregates were no longer evident and eliminated by autophagy [33]. However, it was not clear from those previous results which proteins were modified by ubiquitination. In our study, we also found that GS could increase high-molecular-weight ubiquitinated proteins. Moreover, ubiquitination of Pdx1 and Mafa proteins specifically was observed among the high-molecular-weight proteins. The use of cycloheximide blocked the *de novo* protein synthesis and accelerated the degradation of Pdx1 and Mafa proteins, while MG132 successfully restored GS-induced reduction in protein levels of Pdx1 and Mafa. Hence, these results indicated that the increased ubiquitinated Pdx1 and Mafa caused by GS was eliminated through the ubiquitin-proteasome proteolytic pathway.

As moderate activation of PPARγ with GS treatment was determined in our study, we investigated the downstream effects of this activation. Pre-treatment with TRO or transfection with PPARγ to activate PPARγ activation restored the defective Pdx1 and Mafa protein levels caused by GS. Moreover, TRO inhibited the formation of cellular ubiquitination of Pdx1 and Mafa and normalized their half-lives. The mechanism whereby TRO exerts its protective effects is worthy of further exploration. TZDs including TRO were once widely used to treat type 2 diabetic patients since they were found to increase insulin sensitivity in peripheral tissues [34]. Later, further research determined that TZDs can lead to improvements in islet architecture, insulin content and insulin secretion [20], [23], [35]. Beta-cell specific PPARγ knockout mice exhibit glucose intolerance, impaired GSIS and deficiency in basal Pdx1 expression, as well as loss of sensitivity to the pharmacological effect of TZDs in enhancing Pdx1 expression [36]. Functional PPREs have been identified in the promoters of *Pdx1*, *NKX6.1*, *GLUT2*, *GK* and *Serca2b*, which were confirmed to be physiologically target genes of the nuclear hormone receptor PPARγ in beta-cells [24], [25], [26], [27], [37]. Considering the transcriptional effect of PPARγ, we analyzed the mRNA levels of *Pdx1* and *Mafa* and found that indeed the expression of *Pdx1* but not *Mafa* was regulated by TRO. However, because the mRNA and protein levels of Pdx1 were altered almost simultaneously, we strongly believe that the regulation at the mRNA level by GS treatment contributed little to the Pdx1 protein level. Instead, the activation of PPARγ appeared to modulate Pdx1 and Mafa proteins at the post-trancriptional level by reducing their degradation.

Another distinct finding in our study was the obvious mitochondrial changes induced by GS. Effects on Bcl-2 family members, such as the dramatically decreased *Bcl2l1* and *Bcl2* expression levels, and the morphological changes of mitochondria after GS treatment have previously been observed [11]. Here, we found that TRO pre-treatment could reverse the reduction of *Bcl2l1* and *Bcl2* by GS. Moreover, ectopic expression of Bcl-xl effectively promoted the resumption of Pdx1 and Mafa protein synthesis. Numerous reports have indicated that Bcl2 and Bcl2l1 are key to maintaining the mitochondrial membrane potential and reducing the overproduction of reactive oxygen species (ROS) [38], [39], [40], [41], thereby normalizing mitochondrial function. PPARγ activation has also been observed to benefit the mitochondria in diabetic mice [42], [43]. Some researchers have demonstrated that TRO prevents mitochondrial dysfunction to rescue beta-cell destruction in obese pre-diabetic rats [22]. Others revealed that PPARγ activation up-regulates the Bcl-2 protective pathway to improve mitochondrial function in different cells [44], [45], [46], [47], [48]. Moreover, PPARγ agonists were shown to induce mitochondrial biogenesis in the mouse brain [49], [50]. The existing literature and our results suggest that the protective effect of PPARγ activation against GS-induced damage may be executed by sustaining mitochondrial function via maintaining *Bcl2* and *Bcl2l1* gene expression.

Our previous study revealed that GS could de-phosphorylate Foxo1 and cause its nuclear accumulation, thereby resulting in the decreased Pdx1 protein [9]. Transfection of cells with a dominant-negative Foxo1 mutant successfully prevented the GS-induced reduction in the Pdx1 protein. Moreover, the normalized Pdx1 protein level was found to be independent of the transcriptional activity of Foxo1. A large body of literature has described that Foxo1 and Pdx1 exhibit mutually exclusive patterns of nuclear localization in beta-cells, and constitutive nuclear expression of a mutant Foxo1 is associated with lack of Pdx1 expression [51], [52]. The accumulation of Foxo1 in the nucleus causes nuclear-to-cytoplasmic export of Pdx1 [53]. It was reported that glucose-stimulated activation of AKT and inhibition of GSK3 decrease Pdx1 phosphorylation and delay its degradation [54]. A decrease in glucose level modulates Pdx1 phosphorylation at serines 268 and 272 by the GSK3 kinase and triggers increased turnover of the Pdx1 protein in a GSK3-dependent manner. The role of AKT therefore was determined to inhibit GSK3 kinase activity and protect Pdx1 from GSK3 induced degradation. The author insisted that the stabilizing effect of AKT on Pdx1 is independent of Foxo1-mediated *Pdx1* gene repression. We agree that the transcriptional regulation of Foxo1 on Pdx1 in that situation may not be relevant. However, we reasoned that Foxo1 may function as a mediator to facilitate the export of Pdx1 from the nucleus to the cytoplasm. The aggregated cytoplasmic Pdx1 triggered ubiquitination, and consequently its turnover was accelerated.

Recent studies have documented that PPARγ activation restores islet function through reduction of ER stress and maintenance of euchromatin structure [43]. However, in the present study we examined a marker gene of ER stress, *Ddit3*, and found that its expression was slightly decreased rather than increased. This result demonstrated that ER stress may not have contributed to the observed GS-induced beta-cell dysfunction, and TRO did not exert its beneficial effects through the ER pathway. Pancreatic beta-cells can sense and respond to changing blood glucose levels by using a glucose-sensing apparatus consisting of *Slc2a2* and *Gck* [55]. In diabetic beta-cells, *Slc2a2* and *Gck* gene expressions levels are reduced markedly, and the glucose-sensing capacity is lost, resulting in less insulin secretion under glucose stimulation. We found no significant alterations in *Slc2a2* and *Gck* mRNA levels, suggesting that the glucose-sensing function of the beta-cells did not change in the current study.

In our study, only Pdx1 and Mafa were inhibited by GS, while no changes in Neurod1 or Hnf1a were observed. Activating PPARγ not only restored the protein levels of Pdx1 and Mafa, but also rescued insulin synthesis and secretion. Given the crucial role of Pdx1 and Mafa in maintaining mature beta-cell function, their protein deficiencies can directly influence *insulin* gene expression and protein release [56], [57], [58]. We speculated that the recovery of insulin synthesis and secretion from GS treatment may be associated with the normalization of Pdx1 and Mafa proteins as a result of PPARγ activation.

In conclusion, our study first reveals that PPARγ activation prevents Pdx1 and Mafa proteins from degradation upon exposure to GS and eventually maintains the insulin-producing and secretion phenotype in primary rat pancreatic islets and INS-1 cells. These results present direct evidence that, besides counteracting cell dedifferentiation induced by glucotoxicity and lipotoxicity in pancreatic beta-cells, activation of PPARγ can protect beta-cells from the more severe and sustained toxicity of AGEs.

## Supporting Information

Table S1 **Quantitative real-time PCR primer sequences.**
(DOC)
