# Supplementary material for: PPARγ Activation Attenuates Glycated-Serum Induced Pancreatic Beta-Cell Dysfunction through Enhancing Pdx1 and Mafa Protein Stability
Source: PLoS One. 2013 Feb 12;8(2):e56386. doi: 10.1371/journal.pone.0056386 (PMC3570423; doi:10.1371/journal.pone.0056386)
Supplement: Table S1 — Quantitative real-time PCR primer sequences. (DOC) [file pone.0056386.s001.doc]

| Gene Symbol | Sequence (5’→3’ ) | PCR size (bp) |
| --- | --- | --- |
| *Ins1* | Forward CATAGACCATCAGCA AGCAGG | 181 bp |
|  | Reverse GAAGA AACCACGTTCCCCAC |  |
| *Ins2* | Forward TGTCA AACAGCACCTTTGTGG | 170 bp |
|  | Reverse GTGCCAAGGTCTGAAGGTCAC |  |
| *Pdx1* | Forward GGTATAGCCAGCGAGATGCT | 153 bp |
|  | Reverse TCAGGTGGGAGCCTGATTCT |  |
| *Mafa* | Forward AGCTGGTGTCCATGTCAGTG | 120 bp |
|  | Reverse CGTATTTCTCCTTGTACAGG |  |
| *Bcl2* | Forward CTGAGTACCTGAACCGGCATC | 131 bp |
|  | Reverse GAGCAGCGTCTTCAGAGACAG |  |
| *Bcl2l1* | Forward TGACCACCTAGAGCCTTGGAT | 121 bp |
|  | Reverse CAGGAACCAGCGGTTGAAA |  |
| *Slc2a2* | Forward TCAGCCAGCCTGTGTATGCA | 89 bp |
|  | Reverse TCCACAAGCAGCACAGAGACA |  |
| *Ddit3* | Forward TCCTA CGTGA GGGAC TTGGT | 186 bp |
|  | Reverse ACACA GTCAC AGTTC CGTTG G |  |
| *β-Actin* | Forward AGCCATGTACGTAGCCATCC | 228 bp |
|  | Reverse CTCTCAGCTGTGGTGGTGAA |  |

**Table S1.** Quantitative real-time PCR primer sequences.
